# Modelling the influence of radiosensitivity on development of second primary cancer in out-of-field organs following proton therapy for paediatric cranial cancer

**DOI:** 10.1259/bjr.20230161

**Published:** 2023-09-03

**Authors:** Mikaela Dell'Oro, Puthenparampil Wilson, Michala Short, Dylan Peukert, Eva Bezak

**Affiliations:** 1 Cancer Research Institute, University of South Australia, Adelaide, Australia; 2 Department of Radiation Oncology, Royal Adelaide Hospital, Adelaide, Australia; 3 Australian Centre for Quantitative Imaging, School of Medicine, The University of Western Australia, Perth, Australia; 4 UniSA STEM, University of South Australia, Adelaide, Australia; 5 ARC Training Centre for Integrated Operations for Complex Resources, University of Adelaide, Adelaide, Australia; 6 Department of Physics, University of Adelaide, Adelaide, Australia

## Abstract

**Objective:**

Radiobiological modelling the risks of second primary cancer (SPC) after proton therapy (PT) for childhood cranial cancer remains largely unknown. Organ-specific dose-response risk factors such as radiosensitivity require exploration. This study compared the influence of radiosensitivity data (slope of β_EAR_) on children’s lifetime attributable risks (LAR) of SPC development in out-of-field organs following cranial scattering and scanning PT.

**Methods:**

Out-of-field radiosensitivity parameter estimates for organs (α/β and β_EAR_) were sourced from literature. Physical distances for 13 out-of-field organs were measured and input into Schneider’s SPC model. Sensitivity analyses were performed as a function of radiosensitivity (α/β of 1–10 Gy) and initial slope (β_EAR_) from Japanese/UK data to estimate the influence on the risk of radiation-induced SPC following scattering and scanning PT.

**Results:**

Models showed similar LAR of SPC estimates for age and sex-matched paediatric phantoms, however, for breast there was a significant increase using Japanese β_EAR_ data. For most organs, scattering PT demonstrated a larger risk of LAR for SPC which increased with α/β.

**Conclusion:**

Breast tissue exhibited the highest susceptibility in calculated LAR risk, demonstrating the importance for accurate data input when estimating LAR of SPC.

**Advances in knowledge:**

The findings of this study demonstrated younger female patients undergoing cranial proton therapy have a higher risk of developing second primary cancer of the breast tissue. Long-term multicenter registries are important to improve predictive radiobiological modelling studies of side effects.

## Introduction

As radiotherapy technology advances and cure rates for childhood cancer patients approach 80%, there is increasing survivorship and emphasis placed on long-term side-effects such as second primary cancer (SPC).^1^ Data from 1990 to 2000 age-matched cohorts estimate the life expectancy for children diagnosed with cancer has increased by 9 years compared to previous decades.^2^ As paediatric patients have a longer life expectancy than other patient cohorts, they also have an increased lifetime attributable risk (LAR) of developing SPC post-radiotherapy.^3^ While every effort is being made to increase the length and quality of life of survivors, the type of radiotherapy has been shown to impact life expectancy.^4,5^ Where available, proton therapy (PT) is recommended for paediatric patients with the aim to reduce their risk of long-term health effects such as SPC. A long latency period is required to observe a physical difference in SPC incidence between treatment modalities in paediatric patients.^6^ While the risk of SPC for in-field organs is better understood, there are uncertainties surrounding the SPC risks for out-of-field organs using the latest neutron dose equivalent data.^7^

As long-term follow-up and prospective clinical studies are difficult to conduct, more consideration has been given to modelling studies to determine risk of SPC in children (for latest review see Romero-Expósito et al^8^ and references within). Mathematical prediction of SPC can be somewhat contentious as parameters associated with increased risk are based on historically collected data from high irradiation incidents and adult populations.^9^ The most widely used SPC model developed by Schneider et al^9^ estimates risks using tissue-specific parameters to calculate Excess Absolute Risk (EAR). The initial slope of the dose-risk curve for radiation-induced SPC, referred to as β_EAR_ data, was collected from large epidemiological studies performed on Japanese/Nagasaki Atomic bomb (A-bomb) survivors^10^ or United Kingdom (UK) Hodgkin’s Lymphoma disease survivors.^11^ As the Schneider et al. SPC model depends strongly on the SPC incidence (β_EAR_) of the dose-response curves collected from these adult survivors, the calculated SPC may vary depending on the β_EAR_ source used. There are caveats to the use of both β_EAR_ data sets. The A-bomb surveillance data is influenced by a range of radioisotopes survivors were exposed to and is therefore subject to large uncertainties.^12^ Meanwhile caveats also exist for the UK β_EAR_ data sourced from Hodgkin’s Lymphoma survivors, it is suggested that this patient cohort is genetically more susceptible to developing cancer and thus naturally have a greater chance of developing SPC.^13,14^ This has the potential to overestimate LAR of SPC when applying the EAR to other patient groups.^7^ Therefore, both β_EAR_ datasets have some level of uncertainty and accurate correlation with stray radiation (*e.g.,* neutrons) is problematic. Previously, Brodin et al^15^ used spot-scanning data assuming secondary neutrons are only generated within the patient and the same dose-response assumption for all organs. Building on this, we incorporated worst-case neutron dose equivalents in our modelling to reduce underestimation of the contribution of secondary neutrons.

Additionally, radiosensitivity (represented as α/β ratio in EAR equation) in respect to fractionation of individual out-of-field organs remain largely unknown. By convention a value of 3 Gy is used to represent the α/β ratio of normal tissue, however, this may under or overestimate the true value.^16^ For paediatric patients, the dose-response value may be even more heterogenous for one type of tissue/organ between patients (*i.e.,* interpatient α/β ratio of 1, 3, 5 or 10 Gy). Other parameters affecting the EAR include the sex (*s*) correction factor as well as patient age and age at radiation exposure. All of these parameters used as inputs to calculate the EAR as a function of dose. Currently, it is unknown as to which β_EAR_ data set or α/β ratio to use for modern estimations of SPC in paediatric cranial PT.

### Aims

This study investigated the influence of previously published radiosensitivity data (β_EAR_) on the LAR of SPC for out-of-field organs following contemporary paediatric cranial scattering and scanning PT. Calculations were also performed as a function of α/β ratio for individual out-of-field organs. The sensitivity of the model to a) population data from Japanese/Nagasaki A-bomb survivors compared to UK Hodgkin’s Lymphoma disease survivors and b) the radiosensitivity (α/β ratio) of organs in respect to fractionation was investigated.

## Methods and materials

### Dataset-based phantoms

Ethics approval was obtained from St. Jude Children's Research Hospital and the University of South Australia, ethics code: 202267. CT dataset-based phantoms of six sex matched paediatric patients (5, 9 and 13-years) were sourced from St. Jude Children’s Research Hospital. Detailed proton planning methods were described in our previous publication.^17^

### Second Primary Cancer modelling

**Figure 1.**
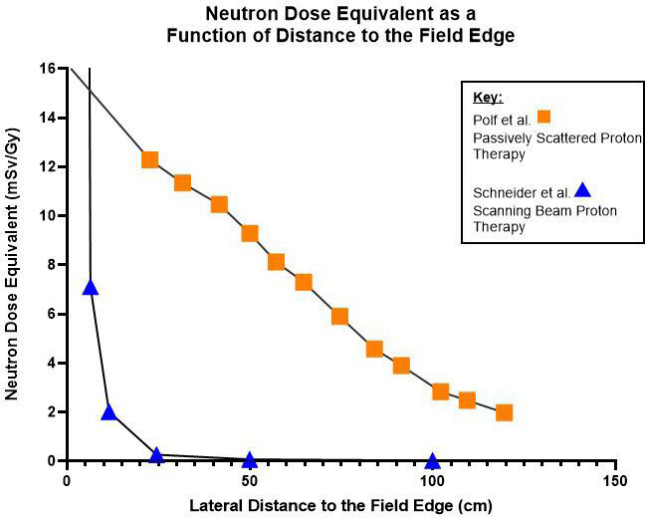
Comparison of neutron dose equivalents used in this study reported for passively scattered (158 MeV) and scanning beam (200 MeV) PT.

The Schneider et al.^9^ SPC model was used for LAR calculations. Neutron dose equivalents for passive scattering PT and scanning PT as a function of distance from the beam edge were adopted from Polf et al^18^ and Schneider et al,^19^ respectively (Figure 1). Although passively scattered PT has nowadays been largely replaced by scanning beam PT, calculating the LARs for the two modalities is still useful. Many of the older treatment facilities still operate scattered PT and, additionally, children treated 10–15 years ago may only now start presenting with SPC. The clinical SPC data for the scanning PT will still take some time to become available.

The neutron dose equivalent to each organ was determined using the total dose delivered to the target volume (54 GyRBE), the organ distances (measured from the inferior edge of the clinical target volume on the CT dataset-based phantoms in the longitudinal axis to the mid-point of the organ and the neutron dose equivalent data presented in Figure 1. The proton plans were planned with an RBE of 1.1 in Eclipse treatment planning software version 13.7 (Varian Medical Systems, Palo Alto, CA). The organ neutron dose equivalents (in mSv per Gy of proton dose delivered) used in the SPC modelling are calculated in the previous publication.^20^ This study builds on the previous work performed in Dell’Oro et al,^20^ looking at the dependence of the input data. The specific organ distances were applied to the corresponding neutron dose equivalent curves (Figure 1) to produce neutron dose equivalents Supplementary Table 1.

Supplementary Material 1.

LAR of SPC induction was calculated using a Schneider et al^9^ analytical model programmed in MATLAB (R2020B) to estimate LAR for SPC. Australian sex-specific life expectancy was sourced from the Australian Bureau of Statistics 2020).^21^ Variables and functions used to calculate LAR of SPC were defined in the previous publication.^20^

The sensitivity values used to model the influence of β_EAR_ and α/β parameters on LAR estimates are listed in Table 1 for 12 out-of-field organs evaluated in this study. Published α [Gy^−1^] values were collated and β [Gy^−2^] values adjusted for α/β ratios of 1, 3, 5 and 10 Gy. Rectum α [Gy^−1^] value was inferred from small intestine and similarly β_EAR_ for reproductive organs (cervix, ovaries and testes) was inferred from rectum.^16^ This is because the structures rapidly repopulate and are classified as an early responding tissue, with similar radiosensitivity. β_EAR_ values of the mean value as well as the upper and lower bounds of the 95% confidence interval (CI) were used to test for significant differences between the cohorts reported by Dores et al.^11^ (UK) and Preston et al.^10^ (Japanese). Data were collated in Excel Microsoft spreadsheets (Excel 2010).

**Table 1.** The β_EAR_ and α/β modifying model parameters used for the LAR estimates in the study

Organ	Japanese β_EAR_			UK β_EAR_			α [Gy^−1^]	β [Gy^−2^]			
	Lower 95% CI	Mean	Upper 95% CI	Lower 95% CI	Mean	Upper 95% CI		α/β = 1	α/β = 3	α/β = 5	α/β = 10
Salivary Gland	0.20	0.56	1.20	0.26	0.73	1.60	0.09	0.09	0.03	0.02	0.01
Thyroid ^*a*^	0.50	1.20	2.20	0.20	0.40	0.80	0.03	0.03	0.01	0.01	0.00
Oesophagus	0.18	0.58	1.10	1.00	3.20	6.10	0.03	0.03	0.01	0.01	0.00
Lung	5.10	7.50	10.00	5.50	8.00	11.00	0.04	0.04	0.01	0.01	0.00
Breast	6.80	9.20	12.00	6.10	8.20	11.00	0.04	0.04	0.01	0.01	0.00
Stomach	6.10	9.50	14.00	3.40	5.20	7.70	0.05	0.05	0.02	0.01	0.00
Liver	0.00	4.30	7.20	0.00	2.40	4.00	0.32	0.32	0.11	0.06	0.03
Colon	4.40	8.00	12.00	4.00	7.40	11.00	0.00	0.00	0.00	0.00	0.00
Small intestine	4.40	8.00	12.00	5.70	10.00	16.00	0.59	0.59	0.20	0.12	0.06
Bladder	1.10	3.20	5.40	1.30	3.80	6.50	0.22	0.22	0.07	0.04	0.02
Rectum	−0.13	0.56	1.40	−0.17	0.73	1.80	0.59	0.59	0.20	0.12	0.06
Reproduction Organs	0.00	0.56	1.90	0.00	0.73	2.50	1.58	1.58	0.53	0.32	0.16

CI, confidence interval.

All are full models except.

a Bell-shaped model/not full model.

### Statistical analysis

LAR data were analysed using GraphPad Prism 7 (V.8) in terms of SPC incidence per 10,000 person years. The radiosensitivity of 12 out-of-field organs was investigated by calculating the LAR of SPC for a range of plausible α/β (Gy) values and pre-collected β_EAR_ values for each age and sex. Organ-specific data were tabulated to demonstrate the influence of α/β for each scattering and scanning treatment modality for β_EAR_ values. Paired t-tests were performed to observe the difference between the populated data. Graphs were plotted for each patient age to compare LAR for treatment modalities by patient sex over a range of α/β (1, 3, 5 and 10 Gy). Sensitivity analysis testing was performed using the 95% CI reported for each β_EAR_. This was then visually plotted to aid identification of potential significant differences between measurements.

## Results

This study estimated the influence of β_EAR_ and α/β on organ-specific LAR of SPC after scattering and scanning beam for clinical scenarios based on six sex and age matched CT dataset-based phantoms. Table 2 shows the influence of β_EAR_ for each scattering and scanning treatment modality across organs. All organ-specific LAR estimates including α/β comparisons can be found in Supplementary Tables 2 and 3.

**Table 2.** Influence of β_EAR_ on organ-specific LAR (per 10,000 person years) for patients undergoing cranial scattering (white) and scanning (grey) PT (α/β = 3 Gy)

	UK β_EAR_						Japanese β_EAR_					
	**5F**	**5M**	**9F**	**9M**	**13F**	**13M**	**5F**	**5M**	**9F**	**9M**	**13F**	**13M**
**Salivary Gland**	75.9	50.5	61.1	48.2	56.2	46.0	58.2	38.7	46.8	37.0	43.1	35.3
	76.8	51.0	74.0	48.9	70.4	46.7	58.9	39.1	56.8	37.5	54.0	35.8
**Thyroid**	184.3	103.7	57.2	49.4	35.9	26.4	552.9	311.2	171.6	148.3	107.8	79.3
	317.4	215.3	258.9	177.5	212.5	145.2	952.3	646.0	776.8	532.4	637.4	435.7
**Breast**	467.4	249.3	156.6	131.1	126.4	85.9	524.4	279.7	175.7	147.1	141.8	96.4
	2600.7	1737.1	2266.1	1540.4	2019.9	1357.7	2917.8	1948.9	2542.4	1728.2	2266.3	1523.3
**Colon**	115.5	82.0	57.9	36.6	36.0	24.9	124.8	88.7	62.6	39.6	38.9	26.9
	5131.3	3375.9	3833.5	2511.7	2990.5	1928.1	5547.3	3649.7	4144.3	2715.3	3232.9	2084.4
**Small Intestine**	130.4	78.3	63.3	50.3	47.5	23.0	104.3	62.6	50.6	40.2	38.0	18.4
	87.6	57.0	78.2	50.2	67.8	48.8	70.1	45.6	62.6	40.2	54.3	39.1
**Rectum**	2.8	1.6	1.3	0.9	0.9	0.6	2.1	1.3	1.0	0.7	0.7	0.5
	2.4	1.7	3.0	1.9	3.6	2.4	1.9	1.3	2.3	1.5	2.7	1.8
**Reproductive Organs**	5.2	2.2	2.6	1.1	1.7	0.6	4.0	1.7	2.0	0.9	1.3	0.5
	2.3	1.5	2.0	1.3	1.7	1.2	1.8	1.2	1.5	1.0	1.3	0.9

Grey shaded rows are scanning PT (Schneider)

White rows are passive scattering PT (Polf)

Supplementary Figures 1-12 demonstrate the influence of radiosensitivity (α/β values and β_EAR_) has on organ-specific LAR of SPC for each age-group. Note that the LAR ranged considerably across organs, *i.e.,* rectum LAR estimates were low (< 100 per 10,000 person years), while more radiosensitive organs such as breast were much higher (> 2,000 per 10,000 person years). The LAR calculations showed that breast in particular demonstrated the highest susceptibility to SPC. The LARs for salivary glands from scanning beam PT were slightly higher than and/or potentially comparable, within the parameter and measurement uncertainties to the LARs from scattering PT. While the reasons for this are not completely clear, it can potentially be attributed to uncertainties in the measured neutron dose equivalents at shorter distances where the scanning beam data display a very sharp gradient closer to the beam edge, resulting in a similar neutron dose equivalents for both techniques. This finding coincides with similar recent reports from Leite et al. ^22^ . The 2021 report demonstrates organs closer to the field (*i.e.,* brainstem) receive almost identical neutron dose for double scattering PT and pencil beam scanning PT.

LARs are higher for reproductive organs for the case of scanning PT. This appears to be linked to the specific radiobiological parameters used for reproductive organs based on Schneider et al ^9^, resulting in EAR and LAR trends that decrease with the increasing radiation dose and perhaps reflect the higher sensitivity of the reproductive organs to radiation damage, where the cells are more likely to be eradicated by radiation rather than mutate and become carcinogenic.

As expected, scattering PT demonstrated higher LAR of SPC for the majority of organs with the exceptions of the salivary gland, small intestine, and reproductive organs. This is likely due to the shape and fall-off of the neutron dose equivalent curves at larger distances. Differences for reproductive organs are minimal across all ages (close to zero). For the salivary gland, the trend appears only for α/β = 1 (Gy). For the small intestine and other distal organs, α/β appears to have less of an influence (if any) on LAR of SPC for scanning PT the further the organ is from the isocentre (Supplementary Tables 2 and 3). Fractionation effects are potentially reduced even at high doses for neutron radiation. While there is some evidence of this for deterministic effects, the impact of fractionation on stochastic effects still needs to be investigated.^23^ This could have been one of the reasons why α/β variation had little impact on LAR of SPC generally. While there was little dependence on α/β in general, this was even less in scanning PT, suggesting a lesser dependence on fractionation at lower irradiation doses. Scanning PT LAR estimates were higher compared to scattering for 5-year-old patients, which demonstrates that for these organs perhaps there is no clear advantage (although overall LAR was small).

**Figure 2.**
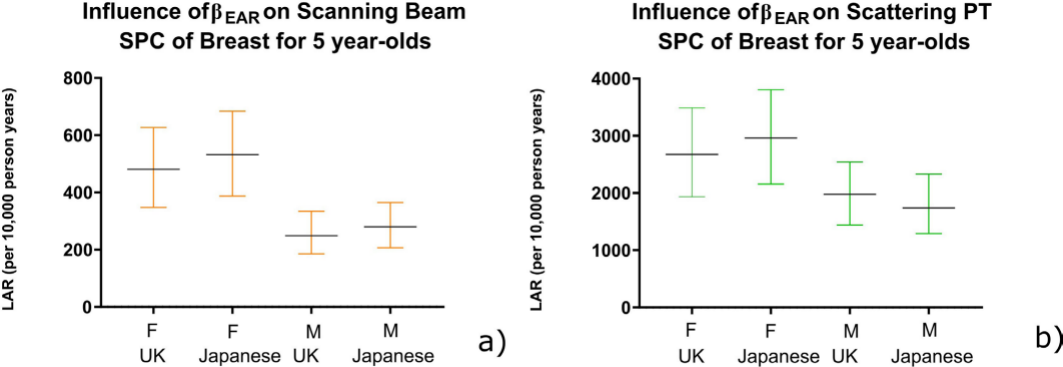
Estimated lifetime attributable risk (per 10,000 person years) of developing second primary cancer of breast for 5-year-old patients following (**a**) scanning and (**b**) scattering PT.

**Figure 3.**
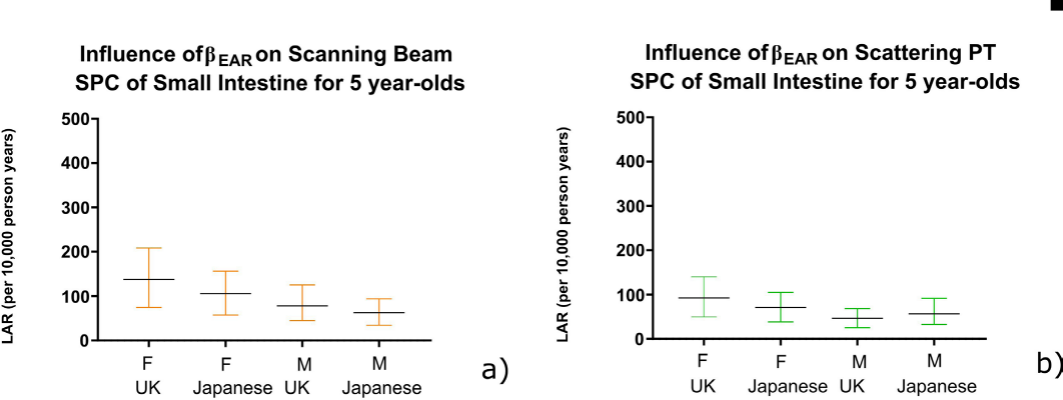
Estimated lifetime attributable risk (per 10,000 person years) of developing second primary cancer of small intestine for 5-year-old patients following (**a**) scanning and (**b**) scattering PT.

In general, β_EAR_ radiosensitivity demonstrated only a small influence on LAR estimates for scanning PT compared to scattering PT. There was a small difference between LAR using both published β_EAR_ data sets, except for breast. The significance of the difference between β_EAR_ datasets was evaluated using the 95% CI range. No significant differences were observed within the error bars, except for breast (*p* = 0.0121) (Figure 2). Breast demonstrated high susceptibility for LAR of SPC, therefore it is important to know which data points (β_EAR_) to use when estimating long-term effects for this organ. All other organs demonstrated no significant difference when investigating upper and lower confidence intervals of β_EAR_. Figure 3 is an example of LAR variation for the small intestine.

The results shown in Supplementary Figures 1-12 demonstrated that using Japanese β_EAR_ data set resulted in higher LAR of SPC than for the UK β_EAR_ data set for the thyroid gland, breast, stomach, liver, colon, bladder and reproductive organs. For all other organs considered including: the salivary glands, oesophagus, lung and small intestine the UK β_EAR_ data set resulted in higher LAR than for the Japanese data set. Scattering PT demonstrated larger risk of LAR for SPC, which increased with α/β for most organs (except for rectum and colon). Meanwhile, scanning beam PT was relatively unchanged with α/β across all organs, indicating some level of robustness.

## Discussion

A range of plausible and reported radiosensitivity values (α/β and β_EAR_) were tested to gauge their influence on LAR of SPC risk for out-of-field organs following paediatric cranial PT treatment. Comparative SPC studies tend to focus comparing LAR estimates of proton (scattering or scanning) compared to photon,^6,24–28^ few studies evaluate the risk of developing radiation-induced SPC as a function of age and/or sex.^7,15,29^ To our knowledge, no study has comprehensively investigated the sensitivity of Schneider’s SPC model and vulnerability to error based on parameter input. The results of this study show the choice of dataset effects the SPC estimates, especially for the scattering proton plans. Limited studies apply both Japanese and reconstructed UK radiotherapy dose response estimates for use with real patient data-based phantoms.^6,15^

Dose-response model parameters from Hodgkin Lymphoma treatments were reconstructed in adult phantoms from plans including large field types (*i.e.,* para-aortic) treated with X-ray doses.^9^ Most of the organs for which doses were calculated were either in-beam, near-beam, or partially-in-beam, rather than out-of-field organs, potentially overestimating the risks of SPC. Yet our calculations still show that for some organs (*e.g.,* thyroid or breast) the Japanese data predict larger risks. Considering that primarily 6 MV X-ray beams were used to deliver doses of 30–40 Gy from mid-1970s to 1994,^11^ there would be little to no neutron production, thus predicting a lower risk of SPC. A-bomb radiation consists of a mixture of γ-rays and neutrons and other particles. As such, the cancer risks obtained from A-bomb survivors from Hiroshima/Nagasaki, may potentially be more applicable to the PT case presented in our study, as they include exposure to neutrons, and may therefore more accurately represent risks in relation to SPC due to neutron out-of-field doses.^30^

Most models assume normal tissue radiosensitivity (α/β) is constant at 3; however, organs such as breast have been shown to have an α/β of up to 5 Gy.^9^ Factors such as α/β and variation in relative biological effectiveness (RBE) remain largely unknown.^31,32^ Paganetti et al^33^ previously investigated proton RBE and interpatient variability of α/β in terms of normal tissue complication probability. Testing a range of plausible α/β values has the propensity to improve the robustness of modelling studies, reducing uncertainty as a large range of heterogeneity is accounted for. Our findings suggest that if organ specific α/β were employed then there would be little influence on LAR of SPC using Schneider’s SPC model. This may have been because the curve of the neutron dose equivalent is flatter as a function of larger distance, especially for scanning PT. However, these results did demonstrate that α/β for organs is more important for scattering PT predictions than for scanning PT.

There is a lack of long-term follow-up data on the incidence and risk of SPC in this population. Long-term studies are necessary to accurately assess the occurrence of second primary cancers and understand their relationship with both scattering and scanning PT.

Range uncertainties and biological effect can introduce uncertainties in predicting LAR of SPC. Every patient is unique, and their response to radiation can vary due to individual factors such as genetic predisposition, dosimetric factors, clinical condition, and lifestyle factors. These individual variations introduce uncertainties when extrapolating risk estimates from general population studies to paediatric patients receiving PT.

The absence of direct comparisons and nature of SPC as a late effect makes it difficult to determine the specific contribution. Ongoing research and advancements in radiobiological modelling, as well as the accumulation of long-term data, will contribute to a better understanding of these uncertainties and help refine the predictions and management strategies.

It is important to know which data source (β_EAR_) is used when estimating LAR of SPC to improve robustness of the model. Studies such as Schneider et al^9^ previously combined standard β_EAR_, however, the accuracy of the values for predicting the risk of SPC was unclear. The 95% CI (range of β_EAR_ values in Table 1) was not very large for breast, lung, rectum (negative lower values), colon, reproductive organs (lower for both was zero), bladder, salivary gland. There was a larger range for oesophagus, stomach, small intestine, liver (lower for both was zero) and thyroid. In fact, the β_EAR_ data set for rectum for both UK/JAP lower estimate was negative. Overall, organ-specific radiosensitivity depended strongly on the initial slope of the dose-response curves (β_EAR_). This modelled sensitivity for some organs such as breast demonstrates susceptibility to error in predicted calculations. When more epidemiological data on SPC becomes available the robustness of the model can be tested, similar to the study by Schneider et al^34^ for risk of breast SPC.

### Limitations

As a theoretical study, there are a several variables which could influence the outcomes of the results. There is a great deal of heterogeneity for normal tissues between patients. Furthermore, individual radiosensitivity factors are difficult to model accurately, adding to the complexity. There are still many uncertainties in the assumptions presented and while the results can be regarded as reasonable predictions, care should be taken in clinical interpretation and application. Absolute risk values are easily affected by uncertainties related to the A-bomb survivor data, although it appears to be better at predicting LAR of SPC for paediatric patients due to the nature of the data collected with a neutron contribution. It is impossible to grasp in a single dose-response parameter to correlate with SPC outcomes. More complex models, which incorporate clinical, genetic, and dosimetric factors into a multifactorial SPC models, are required. Such models would allow for an individual patient-based risk assessment.^35^

Although models are useful, they are not perfect, and caution should be taken when interpreting the results of our study as several assumptions have been made in the simplification of a biologically complex issue. Until epidemiological data analysing the rate of SPC in organ-specific circumstances becomes available for paediatric patients, inherent uncertainties will exist.

Improved spot scanning techniques have been developed which may not be reflected by the current study. Studies of the neutron dose equivalent distributions for the latest techniques are scarce and often do not provide data at the distances relevant for paediatric treatments.^36^ The availability of such data would enable future predictions of LAR of SPC to better reflect the latest treatment techniques. A theoretical study by Schneider et al^37^ calculated neutron doses of 0.5 mGy to near-field organs of breast and 1.2 mGy to thyroid (per 1.6 GyRBE fraction) at similar proton energies (138 MeV) during a paediatric craniospinal treatment. However, they do not provide the neutron dose equivalents, which makes a direct comparison with data used in this work difficult. Neutron spectra (and their variations with distance) must be known to correctly apply neutron radiation weighting factors in order to calculate the corresponding neutron dose equivalents. In summary, there is an absence of patient-specific measurements and calculations of neutron doses as well as the impact of spectral variations on risk calculations in patients of different sizes and ages.

### Future considerations

Single and multi-institutional groups are awaiting similar long-term epidemiological data for scanning PT to analyse the impact of SPC.^38^ Modelled risks are continually being investigated, supplemented, and expanded in the literature in accordance with advancing treatment planning system capabilities and clinical technology advancements. In the future, the late effect of SPC from stray radiation could potentially be calculated alongside other late side effects estimated from the treatment planning system (*i.e.,* Monte Carlo simulations). The results can then be validated against clinical data as it becomes available. Identifying risk factors for adverse effects following PT could aid the generation of treatment guidelines.

## Conclusion

This study investigated the influence of radiosensitivity parameters on the risk of radiation-induced SPC through the evaluation of LAR calculations. In general, there little difference between published Japanese and UK dose-response data (β_EAR_), except for breast. Breast demonstrated highest susceptibility in predicted calculations, demonstrating the importance for accurate data input when estimating LAR of SPC. Organ-specific radiosensitivity (α/β ratio) demonstrated a small influence on LAR estimates for scanning PT compared to scattering PT.
