# Supplementary material for: Modelling the influence of radiosensitivity on development of second primary cancer in out-of-field organs following proton therapy for paediatric cranial cancer
Source: Br J Radiol. 2023 Sep 3;96(1150):20230161. doi: 10.1259/bjr.20230161 (PMC10546440; doi:10.1259/bjr.20230161)
Supplement: Supplementary Material 1. [file bjr.20230161.suppl-01.docx]

**Supplementary Table 1.** *Neutron dose equivalent (mSv/Gy) calculated using the measured distances (determined from CT) applied to the corresponding neutron dose equivalent curves from Polf et al. ^18^ (passive scattered PT) and Schneider et al ^19^(scanning beam PT).*

| Organ | Neutron Dose Equivalent (mSv/Gy) | | | | | | | | | | | |
| --- | --- | --- | --- | --- | --- | --- | --- | --- | --- | --- | --- | --- |
|  | 5-year-old | | | | 9-year-old | | | | 13-year-old | | | |
|  | Female | | Male | | Female | | Male | | Female | | Male | |
|  | Schneider  (scanning) | Polf  (scattering) | Schneider  (scanning) | Polf  (scattering) | Schneider  (scanning) | Polf  (scattering) | Schneider  (scanning) | Polf  (scattering) | Schneider  (scanning) | Polf  (scattering) | Schneider  (scanning) | Polf  (scattering) |
| Salivary Gland | 16.3 | 15.1 | 24.3 | 15.2 | 4.9 | 14.7 | 12.2 | 15.0 | 4.6 | 14.6 | 10.7 | 15.0 |
| Thyroid | 8.7 | 14.9 | 5.7 | 14.7 | 2.4 | 14.2 | 3.0 | 14.4 | 1.6 | 13.9 | 1.8 | 14.0 |
| Oesophagus | 10.7 | 15.0 | 6.1 | 14.8 | 2.5 | 14.3 | 3.6 | 14.5 | 2.4 | 14.2 | 2.1 | 14.1 |
| Lung | 4.6 | 14.6 | 3.4 | 14.4 | 1.5 | 13.8 | 2.0 | 14.1 | 1.2 | 13.7 | 1.4 | 13.8 |
| Breast | 2.4 | 14.2 | 1.8 | 14.0 | 0.8 | 13.2 | 0.9 | 13.4 | 0.7 | 13.0 | 0.7 | 13.1 |
| Stomach | 0.6 | 12.8 | 0.5 | 12.6 | 0.2 | 11.4 | 0.3 | 12.0 | 0.2 | 11.4 | 0.2 | 11.2 |
| Liver | 0.8 | 13.2 | 0.6 | 12.9 | 0.3 | 11.8 | 0.4 | 12.3 | 0.3 | 11.9 | 0.2 | 11.1 |
| Colon | 0.4 | 12.2 | 0.4 | 12.4 | 0.2 | 11.4 | 0.2 | 11.3 | 0.2 | 10.8 | 0.2 | 11.1 |
| Small intestine | 0.4 | 12.2 | 0.3 | 11.8 | 0.2 | 10.9 | 0.3 | 11.6 | 0.2 | 10.7 | 0.1 | 10.0 |
| Bladder | 0.2 | 11.0 | 0.2 | 10.7 | 0.1 | 9.3 | 0.1 | 9.3 | 0.1 | 7.8 | 0.1 | 8.2 |
| Rectum | 0.1 | 10.3 | 0.1 | 10.2 | 0.1 | 8.7 | 0.1 | 8.7 | 0.1 | 8.0 | 0.0 | 7.0 |
| Reproductive Organs | 0.1 | 10.2 | 0.1 | 8.9 | 0.1 | 8.6 | 0.0 | 7.1 | 0.1 | 7.6 | 0.0 | 5.2 |
| **Key:** Reproductive organ is testes for males and ovaries for females. | | | | | | | | | | | | |

**Supplementary Table 2. Influence of α/β on organ-specific LAR (per 10,000 person years) for patients undergoing cranial scattering (white) and scanning (grey) PT using UK** **β_EAR_.**

|  | **α/β= 1** | | | | | | **α/β= 3** | | | | | | **α/β= 5** | | | | | | **α/β= 10** | | | | | |
| --- | --- | --- | --- | --- | --- | --- | --- | --- | --- | --- | --- | --- | --- | --- | --- | --- | --- | --- | --- | --- | --- | --- | --- | --- |
|  | **5F** | **5M** | **9F** | **9M** | **13F** | **13M** | **5F** | **5M** | **9F** | **9M** | **13F** | **13M** | **5F** | **5M** | **9F** | **9M** | **13F** | **13M** | **5F** | **5M** | **9F** | **9M** | **13F** | **13M** |
| **Salivary Gland** | 69.4 | 46.2 | 58.4 | 44.1 | 54.0 | 42.0 | 75.9 | 50.5 | 61.1 | 48.2 | 56.2 | 46.0 | 77.3 | 51.4 | 61.6 | 49.1 | 56.6 | 46.8 | 78.3 | 52.1 | 62.0 | 49.7 | 57.0 | 47.4 |
|  | 55.4 | 36.6 | 54.2 | 35.4 | 51.6 | 33.8 | 76.8 | 51.0 | 74.0 | 48.9 | 70.4 | 46.7 | 82.4 | 54.7 | 79.1 | 52.4 | 75.2 | 50.0 | 86.9 | 57.7 | 83.1 | 55.2 | 79.1 | 52.7 |
| **Thyroid** | 179.1 | 101.9 | 57.0 | 49.2 | 35.9 | 26.4 | 184.3 | 103.7 | 57.2 | 49.4 | 35.9 | 26.4 | 185.4 | 104.1 | 57.2 | 49.5 | 35.9 | 26.5 | 186.2 | 104.4 | 57.2 | 49.5 | 35.9 | 26.5 |
|  | 272.8 | 185.6 | 225.6 | 154.1 | 186.0 | 127.0 | 317.4 | 215.3 | 258.9 | 177.5 | 212.5 | 145.2 | 327.3 | 221.9 | 266.2 | 182.6 | 218.3 | 149.2 | 334.9 | 227.0 | 271.8 | 186.6 | 222.7 | 152.3 |
| **Oesophagus** | 157.0 | 90.2 | 63.6 | 57.7 | 62.0 | 36.9 | 159.6 | 91.3 | 63.7 | 58.0 | 62.1 | 37.0 | 160.1 | 91.6 | 63.8 | 58.0 | 62.2 | 37.0 | 160.5 | 91.8 | 63.8 | 58.1 | 62.2 | 37.0 |
|  | 540.5 | 358.1 | 530.6 | 361.2 | 543.8 | 363.3 | 579.0 | 382.9 | 563.7 | 384.6 | 577.2 | 385.2 | 587.5 | 388.4 | 570.9 | 389.7 | 584.5 | 390.0 | 594.0 | 392.6 | 576.5 | 393.7 | 590.1 | 393.7 |
| **Lung** | 183.5 | 96.0 | 71.7 | 63.7 | 70.1 | 46.0 | 185.4 | 96.5 | 71.8 | 63.8 | 70.2 | 46.1 | 185.8 | 96.6 | 71.8 | 63.8 | 70.2 | 46.1 | 186.1 | 96.7 | 71.8 | 63.9 | 70.2 | 46.1 |
|  | 777.0 | 506.7 | 787.7 | 527.6 | 838.3 | 545.1 | 848.8 | 552.5 | 850.8 | 572.3 | 905.5 | 587.4 | 864.7 | 562.7 | 864.7 | 582.1 | 920.3 | 596.7 | 877.0 | 570.6 | 875.4 | 589.7 | 931.7 | 603.9 |
| **Breast** | 465.7 | 248.8 | 156.6 | 131.1 | 126.3 | 85.9 | 467.4 | 249.3 | 156.6 | 131.1 | 126.4 | 85.9 | 467.7 | 249.3 | 156.6 | 131.2 | 126.4 | 85.9 | 468.0 | 249.4 | 156.6 | 131.2 | 126.4 | 85.9 |
|  | 2196.9 | 1474.4 | 1967.6 | 1326.0 | 1758.3 | 1183.0 | 2600.7 | 1737.1 | 2266.1 | 1540.4 | 2019.9 | 1357.7 | 2689.7 | 1794.8 | 2330.7 | 1587.0 | 2076.5 | 1395.5 | 2759.4 | 1840.0 | 2381.1 | 1623.5 | 2120.6 | 1424.9 |
| **Stomach** | 28.1 | 16.9 | 14.4 | 11.8 | 12.7 | 8.2 | 28.2 | 16.9 | 14.4 | 11.8 | 12.7 | 8.2 | 28.2 | 16.9 | 14.4 | 11.8 | 12.7 | 8.2 | 28.2 | 16.9 | 14.4 | 11.8 | 12.7 | 8.2 |
|  | 67.0 | 45.1 | 72.1 | 48.0 | 76.0 | 51.0 | 92.5 | 61.9 | 96.1 | 64.4 | 99.4 | 66.8 | 93.0 | 62.2 | 96.5 | 64.7 | 99.8 | 67.1 | 93.3 | 62.5 | 96.8 | 64.9 | 100.1 | 67.3 |
| **Liver** | 23.3 | 12.1 | 10.0 | 8.3 | 9.8 | 4.6 | 23.4 | 12.1 | 10.0 | 8.3 | 9.8 | 4.6 | 23.4 | 12.1 | 10.0 | 8.3 | 9.8 | 4.6 | 23.4 | 12.1 | 10.0 | 8.3 | 9.8 | 4.6 |
|  | 34.2 | 22.9 | 35.1 | 22.7 | 34.0 | 23.3 | 43.7 | 29.1 | 44.3 | 28.8 | 42.9 | 29.2 | 46.4 | 30.8 | 46.8 | 30.5 | 45.3 | 30.8 | 48.6 | 32.3 | 48.9 | 31.9 | 47.4 | 32.1 |
| **Colon** | 115.5 | 82.0 | 57.9 | 36.6 | 36.0 | 24.9 | 115.5 | 82.0 | 57.9 | 36.6 | 36.0 | 24.9 | 115.5 | 82.0 | 57.9 | 36.6 | 36.0 | 24.9 | 115.5 | 82.0 | 57.9 | 36.6 | 36.0 | 24.9 |
|  | 5121.0 | 3369.0 | 3827.1 | 2507.5 | 2986.1 | 1925.2 | 5131.3 | 3375.9 | 3833.5 | 2511.7 | 2990.5 | 1928.1 | 5133.4 | 3377.4 | 3834.8 | 2512.5 | 2991.4 | 1928.7 | 5135.0 | 3378.4 | 3835.8 | 2513.2 | 2992.0 | 1929.1 |
| **Small Intestine** | 130.2 | 78.2 | 63.3 | 50.2 | 47.5 | 23.0 | 130.4 | 78.3 | 63.3 | 50.3 | 47.5 | 23.0 | 130.4 | 78.3 | 63.3 | 50.3 | 47.5 | 23.0 | 130.4 | 78.3 | 63.3 | 50.3 | 47.5 | 23.0 |
|  | 69.9 | 45.6 | 62.1 | 39.9 | 53.6 | 38.1 | 87.6 | 57.0 | 78.2 | 50.2 | 67.8 | 48.8 | 92.6 | 60.3 | 82.9 | 53.2 | 71.9 | 51.8 | 96.9 | 63.1 | 86.8 | 55.7 | 75.4 | 54.4 |
| **Rectum** | 2.8 | 1.6 | 1.3 | 0.9 | 0.9 | 0.6 | 2.8 | 1.6 | 1.3 | 0.9 | 0.9 | 0.6 | 2.8 | 1.6 | 1.3 | 0.9 | 0.9 | 0.6 | 3.0 | 1.8 | 1.4 | 1.0 | 0.9 | 0.6 |
|  | 1.6 | 1.1 | 1.8 | 1.2 | 2.2 | 1.5 | 2.4 | 1.7 | 3.0 | 1.9 | 3.6 | 2.4 | 2.2 | 1.5 | 2.7 | 1.7 | 3.2 | 2.1 | 92.9 | 61.8 | 85.4 | 57.5 | 77.5 | 51.8 |
| **Bladder** | 13.3 | 8.2 | 6.5 | 4.6 | 5.0 | 2.7 | 13.3 | 8.2 | 6.5 | 4.6 | 5.0 | 2.7 | 13.3 | 8.2 | 6.5 | 4.6 | 5.0 | 2.7 | 13.3 | 8.2 | 6.5 | 4.6 | 5.0 | 2.7 |
|  | 69.2 | 48.1 | 95.0 | 61.1 | 102.7 | 75.8 | 104.3 | 71.4 | 126.2 | 82.3 | 130.2 | 92.6 | 113.6 | 77.5 | 133.7 | 87.5 | 136.6 | 96.4 | 121.1 | 82.5 | 139.7 | 91.6 | 141.6 | 99.4 |
| **Reproductive Organs** | 5.1 | 2.2 | 2.6 | 1.1 | 1.7 | 0.6 | 5.2 | 2.2 | 2.6 | 1.1 | 1.7 | 0.6 | 5.2 | 2.2 | 2.6 | 1.1 | 1.7 | 0.6 | 5.2 | 2.2 | 2.6 | 1.1 | 1.7 | 0.6 |
|  | 1.9 | 1.3 | 1.7 | 1.2 | 1.5 | 1.0 | 2.3 | 1.5 | 2.0 | 1.3 | 1.7 | 1.2 | 2.4 | 1.6 | 2.1 | 1.4 | 1.8 | 1.2 | 2.5 | 1.6 | 2.1 | 1.4 | 1.8 | 1.2 |
| **Key:** Grey shaded rows are scanning PT (Schneider) and white rows are passive scattering PT (Polf). | | | | | | | | | | | | | | | | | | | | | | | | |

**Supplementary Table 3. Influence of α/β on organ-specific LAR (per 10,000 person years) for patients undergoing cranial scattering (white) and scanning (grey) PT using Japanese β_EAR_.**

|  | **α/β= 1** | | | | | | **α/β= 3** | | | | | | **α/β= 5** | | | | | | **α/β= 10** | | | | | |
| --- | --- | --- | --- | --- | --- | --- | --- | --- | --- | --- | --- | --- | --- | --- | --- | --- | --- | --- | --- | --- | --- | --- | --- | --- |
|  | **5F** | **5M** | **9F** | **9M** | **13F** | **13M** | **5F** | **5M** | **9F** | **9M** | **13F** | **13M** | **5F** | **5M** | **9F** | **9M** | **13F** | **13M** | **5F** | **5M** | **9F** | **9M** | **13F** | **13M** |
| **Salivary Gland** | 53.2 | 35.4 | 44.8 | 33.8 | 41.4 | 32.3 | 58.2 | 38.7 | 46.8 | 37.0 | 43.1 | 35.3 | 59.3 | 39.4 | 47.2 | 37.6 | 43.4 | 35.9 | 60.1 | 40.0 | 47.6 | 38.2 | 43.7 | 36.4 |
|  | 42.5 | 28.1 | 41.5 | 27.1 | 39.6 | 25.9 | 58.9 | 39.1 | 56.8 | 37.5 | 54.0 | 35.8 | 63.2 | 42.0 | 60.6 | 40.2 | 57.7 | 38.4 | 66.7 | 44.3 | 63.8 | 42.4 | 60.7 | 40.4 |
| **Thyroid** | 537.4 | 305.7 | 171.0 | 147.6 | 107.6 | 79.2 | 552.9 | 311.2 | 171.6 | 148.3 | 107.8 | 79.3 | 556.1 | 312.3 | 171.7 | 148.5 | 107.8 | 79.4 | 558.5 | 313.2 | 171.7 | 148.6 | 107.8 | 79.4 |
|  | 818.3 | 556.7 | 676.9 | 462.3 | 557.9 | 380.9 | 952.3 | 646.0 | 776.8 | 532.4 | 637.4 | 435.7 | 981.9 | 665.7 | 798.7 | 547.8 | 654.8 | 447.7 | 1004.8 | 680.9 | 815.5 | 559.7 | 668.1 | 456.9 |
| **Oesophagus** | 28.5 | 16.3 | 11.5 | 10.5 | 11.2 | 6.7 | 28.9 | 16.6 | 11.6 | 10.5 | 11.3 | 6.7 | 29.0 | 16.6 | 11.6 | 10.5 | 11.3 | 6.7 | 29.1 | 16.6 | 11.6 | 10.5 | 11.3 | 6.7 |
|  | 98.0 | 64.9 | 96.2 | 65.5 | 98.6 | 65.8 | 104.9 | 69.4 | 102.2 | 69.7 | 104.6 | 69.8 | 106.5 | 70.4 | 103.5 | 70.6 | 105.9 | 70.7 | 107.7 | 71.2 | 104.5 | 71.4 | 107.0 | 71.4 |
| **Lung** | 172.0 | 90.0 | 67.2 | 59.7 | 65.7 | 43.2 | 173.8 | 90.5 | 67.3 | 59.8 | 65.8 | 43.2 | 174.2 | 90.6 | 67.3 | 59.8 | 65.8 | 43.2 | 174.5 | 90.7 | 67.3 | 59.9 | 65.8 | 43.2 |
|  | 728.5 | 475.0 | 738.5 | 494.6 | 785.9 | 511.1 | 795.7 | 518.0 | 797.7 | 536.5 | 848.9 | 550.7 | 810.6 | 527.5 | 810.7 | 545.7 | 862.7 | 559.4 | 822.2 | 534.9 | 820.7 | 552.9 | 873.4 | 566.1 |
| **Breast** | 522.5 | 279.1 | 175.6 | 147.1 | 141.7 | 96.3 | 524.4 | 279.7 | 175.7 | 147.1 | 141.8 | 96.4 | 524.8 | 279.8 | 175.7 | 147.2 | 141.8 | 96.4 | 525.1 | 279.8 | 175.7 | 147.2 | 141.8 | 96.4 |
|  | 2464.9 | 1654.2 | 2207.6 | 1487.7 | 1972.7 | 1327.3 | 2917.8 | 1948.9 | 2542.4 | 1728.2 | 2266.3 | 1523.3 | 3017.7 | 2013.7 | 2614.9 | 1780.6 | 2329.7 | 1565.6 | 3096.0 | 2064.4 | 2671.4 | 1821.5 | 2379.2 | 1598.6 |
| **Stomach** | 51.4 | 30.8 | 26.3 | 21.5 | 23.2 | 15.0 | 51.5 | 30.9 | 26.3 | 21.6 | 23.2 | 15.0 | 51.5 | 30.9 | 26.3 | 21.6 | 23.2 | 15.0 | 51.5 | 30.9 | 26.3 | 21.6 | 23.2 | 15.0 |
|  | 122.4 | 82.4 | 131.8 | 87.7 | 138.8 | 93.1 | 168.9 | 113.1 | 175.5 | 117.6 | 181.6 | 122.0 | 169.8 | 113.7 | 176.3 | 118.1 | 182.3 | 122.5 | 170.5 | 114.1 | 176.9 | 118.6 | 182.9 | 122.9 |
| **Liver** | 41.8 | 21.7 | 17.8 | 14.9 | 17.6 | 8.3 | 41.9 | 21.7 | 17.8 | 14.9 | 17.6 | 8.3 | 41.9 | 21.7 | 17.9 | 14.9 | 17.6 | 8.3 | 42.0 | 21.7 | 17.9 | 14.9 | 17.6 | 8.3 |
|  | 61.3 | 41.0 | 63.0 | 40.8 | 60.9 | 41.8 | 78.3 | 52.1 | 79.4 | 51.6 | 76.9 | 52.4 | 83.1 | 55.3 | 83.9 | 54.6 | 81.2 | 55.2 | 87.0 | 57.9 | 87.6 | 57.1 | 84.8 | 57.6 |
| **Colon** | 124.8 | 88.7 | 62.6 | 39.6 | 38.9 | 26.9 | 124.8 | 88.7 | 62.6 | 39.6 | 38.9 | 26.9 | 124.8 | 88.7 | 62.6 | 39.6 | 38.9 | 26.9 | 124.8 | 88.7 | 62.6 | 39.6 | 38.9 | 26.9 |
|  | 5536.2 | 3642.2 | 4137.4 | 2710.8 | 3228.2 | 2081.3 | 5547.3 | 3649.7 | 4144.3 | 2715.3 | 3232.9 | 2084.4 | 5549.6 | 3651.2 | 4145.8 | 2716.2 | 3233.9 | 2085.1 | 5551.3 | 3652.4 | 4146.8 | 2716.9 | 3234.6 | 2085.5 |
| **Small Intestine** | 104.2 | 62.6 | 50.6 | 40.2 | 38.0 | 18.4 | 104.3 | 62.6 | 50.6 | 40.2 | 38.0 | 18.4 | 104.3 | 62.6 | 50.6 | 40.2 | 38.0 | 18.4 | 104.3 | 62.6 | 50.6 | 40.2 | 38.0 | 18.4 |
|  | 56.0 | 36.5 | 49.7 | 31.9 | 42.9 | 30.5 | 70.1 | 45.6 | 62.6 | 40.2 | 54.3 | 39.1 | 74.1 | 48.2 | 66.3 | 42.5 | 57.5 | 41.5 | 77.5 | 50.5 | 69.5 | 44.6 | 60.3 | 43.5 |
| **Rectum** | 2.1 | 1.3 | 1.0 | 0.7 | 0.7 | 0.5 | 2.1 | 1.3 | 1.0 | 0.7 | 0.7 | 0.5 | 2.1 | 1.3 | 1.0 | 0.7 | 0.7 | 0.5 | 2.3 | 1.3 | 1.1 | 0.7 | 0.7 | 0.5 |
|  | 1.2 | 0.8 | 1.4 | 0.9 | 1.7 | 1.1 | 1.9 | 1.3 | 2.3 | 1.5 | 2.7 | 1.8 | 1.7 | 1.1 | 2.1 | 1.3 | 2.4 | 1.6 | 71.3 | 47.4 | 65.5 | 44.1 | 59.4 | 39.7 |
| **Bladder** | 11.2 | 6.9 | 5.5 | 3.9 | 4.2 | 2.3 | 11.2 | 6.9 | 5.5 | 3.9 | 4.2 | 2.3 | 11.2 | 6.9 | 5.5 | 3.9 | 4.2 | 2.3 | 11.2 | 6.9 | 5.5 | 3.9 | 4.2 | 2.3 |
|  | 58.3 | 40.5 | 80.0 | 51.5 | 86.5 | 63.8 | 87.8 | 60.2 | 106.3 | 69.3 | 109.6 | 78.0 | 95.6 | 65.3 | 112.6 | 73.7 | 115.0 | 81.2 | 102.0 | 69.5 | 117.6 | 77.1 | 119.2 | 83.7 |
| **Reproductive Organs** | 4.0 | 1.7 | 2.0 | 0.9 | 1.3 | 0.5 | 4.0 | 1.7 | 2.0 | 0.9 | 1.3 | 0.5 | 4.0 | 1.7 | 2.0 | 0.9 | 1.3 | 0.5 | 4.0 | 1.7 | 2.0 | 0.9 | 1.3 | 0.5 |
|  | 1.5 | 1.0 | 1.3 | 0.9 | 1.1 | 0.8 | 1.8 | 1.2 | 1.5 | 1.0 | 1.3 | 0.9 | 1.9 | 1.2 | 1.6 | 1.0 | 1.3 | 0.9 | 1.9 | 1.3 | 1.6 | 1.1 | 1.4 | 0.9 |
| **Key:** Grey shaded rows are scanning PT (Schneider) and white rows are passive scattering PT (Polf). | | | | | | | | | | | | | | | | | | | | | | | | |

a)

b)

c)

**Supplementary Figure 1**. Estimated lifetime attributable risk (per 10,000 person years) of developing second primary cancer of the salivary gland for a) 5-year-olds, b) 9-year-olds and c) 13-year-olds.

a)

b)

c)

**Supplementary Figure 2**. Estimated lifetime attributable risk (per 10,000 person years) of developing second primary cancer of the thyroid for a) 5-year-olds, b) 9-year-olds and c) 13-year-olds.

a)

b)

c)

**Supplementary Figure 3**. Estimated lifetime attributable risk (per 10,000 person years) of developing second primary cancer of oesophagus for a) 5-year-olds, b) 9-year-olds and c) 13-year-olds.

a)

b)

c)

**Supplementary Figure 4**. Estimated lifetime attributable risk (per 10,000 person years) of developing second primary cancer of the lung for a) 5-year-olds, b) 9-year-olds and c) 13-year-olds.

a)

b)

c)

**Supplementary Figure 5**. Estimated lifetime attributable risk (per 10,000 person years) of developing second primary cancer of the breast for a) 5-year-olds, b) 9-year-olds and c) 13-year-olds.

a)

b)

c)

**Supplementary Figure 6**. Estimated lifetime attributable risk (per 10,000 person years) of developing second primary cancer of the stomach for a) 5-year-olds, b) 9-year-olds and c) 13-year-olds.

a)

b)

c)

**Supplementary Figure 7**. Estimated lifetime attributable risk (per 10,000 person years) of developing second primary cancer of the liver for a) 5-year-olds, b) 9-year-olds and c) 13-year-olds.

a)

b)

c)

**Supplementary Figure 8**. Estimated lifetime attributable risk (per 10,000 person years) of developing second primary cancer of the colon for a) 5-year-olds, b) 9-year-olds and c) 13-year-olds.

a)

b)

c)

**Supplementary Figure** **9**. Estimated lifetime attributable risk (per 10,000 person years) of developing second primary cancer of the small intestine for a) 5-year-olds, b) 9-year-olds and c) 13-year-olds.

a)

b)

c)

**Supplementary Figure 10**. Estimated lifetime attributable risk (per 10,000 person years) of developing second primary cancer of the rectum for a) 5-year-olds, b) 9-year-olds and c) 13-year-olds.

a)

b)

c)

**Supplementary Figure 11**. Estimated lifetime attributable risk (per 10,000 person years) of developing second primary cancer of the bladder for a) 5-year-olds, b) 9-year-olds and c) 13-year-olds.

a)

b)

c)

**Supplementary Figure 12**. Estimated lifetime attributable risk (per 10,000 person years) of developing second primary cancer of the reproductive organs for a) 5-year-olds, b) 9-year-olds and c) 13-year-olds.
